# Sonographic double patterns in hepatic alveolar echinococcosis according to the echinococcosis multilocularis ultrasound classification

**DOI:** 10.1007/s00436-025-08593-y

**Published:** 2025-11-15

**Authors:** Nele Hergesell, Dennis Skotnik, Wolfgang Kratzer

**Affiliations:** Department of Internal Medicine I, Ulm University Hospital, Ultrasound Unit Albert-Einstein-Allee 23, 89081 Ulm, Germany

**Keywords:** Alveolar echinococcosis, Echinococcus multilocularis, Double pattern, Ultrasonography, EMUC-US-Classification

## Abstract

Alveolar echinococcosis (AE) is a rare but severe zoonosis caused by*Echinococcus multilocularis* that predominantly affects the liver. While previous studies have focused on single ultrasound patterns, the significance of hepatic double pattern lesions is not yet fully understood. The aim of this study is to investigate the prevalence, characteristics and combinations of sonographic double patterns in AE. Based on data from Germany’s national echinococcosis database (n=825),40 patients with AE and confirmed double pattern were analysed retrospectively. Inclusion criteria were patients with at least two hepatic lesions of different patterns on reference ultrasound (US). US images were evaluated using the Echinococcosis Multilocularis Ulm Classification (EMUC)-US classification, with the analysis conducted by independent, blinded sonographers. Overall, a double pattern was detected in 4.8 % of patients (n=40). Ten different pattern combinations between two different patterns were observed, with the hailstorm and ossification patterns being the most common combination (52.2 %). Pattern combinations of up to four patterns were found in a single patient. A statistically significant correlation between the pattern combination and lesion activity, as determined by ^18^FDG-PET/CT and serological markers, could not be demonstrated. Sonographic double pattern manifestations of AE are rare. Currently, the occurrence of specific patterns and their combinations does not correlate with lesion activity. Further studies with larger sample sizes are needed to better understand the significance of double patterns and their potential prognostic value.

## Introduction

Alveolar echinococcosis (AE) is a rare, yet potentially lethal zoonosis caused by the larval stage of the fox tapeworm *Echinococcus multilocularis*. The metacestodes (larvae) primarily manifest as hepatic lesions in the human false intermediate host. With an infiltrative and destructive growth pattern, they mimic malignant tumours, inducing chronic inflammation in the host tissue (Wen et al. 2019).

AE is primarily endemic to the Northern Hemisphere, with Southwestern Germany identified as a high-risk region for some time (Schmidberger et al. 2018). Recent reports indicate increasing prevalence worldwide, as well as the expansion beyond the classic endemic areas (Baumann et al. 2019; Casulli et al. 2025). The only curative treatment for AE is complete surgical removal of the infected tissue, followed by a two-year course of anthelmintic treatment. In the case of unresectable findings at the time of diagnosis, long-term treatment with benzimidazoles (BMZ), is required (Brunetti et al. 2010).

The diagnosis of AE relies on a combination of clinical evaluation, imaging modalities, and serological tests. In recent years, imaging diagnostics have become increasingly important compared to serological diagnostics in AE (Calame et al. 2022; Kratzer et al. 2022; Tao et al. 2024). B-scan ultrasonography (US), with its rapid feasibility, widespread availability, and lack of radiation exposure, has become the most common primary diagnostic tool for both initial diagnosis and follow-up. Complementary imaging techniques, such as contrast-enhanced ultrasound (CEUS), computed tomography (CT), magnetic resonance imaging (MRI), and ^18^Fluorodeoxyglucose positron emission tomography (^18^FDG-PET), are used for differential diagnosis and pre-operative workup (Liu et al. 2014). Among these, ^18^FDG/PET-CT remains the most reliable non-invasive method for assessing parasitic activity (Yangdan et al. 2021).

Given its long asymptomatic latency period of up to 15 years, AE is frequently diagnosed during ultrasound assessment of hepatic incidentalomas or the evaluation of non-specific abdominal pain (Joos et al. 2023). However, the polymorphic appearance of AE lesions on US, along with their resemblance to both benign and malignant liver lesions, poses a persistent diagnostic challenge in clinical practice (Kratzer et al. 2022).

**Fig. 1:**
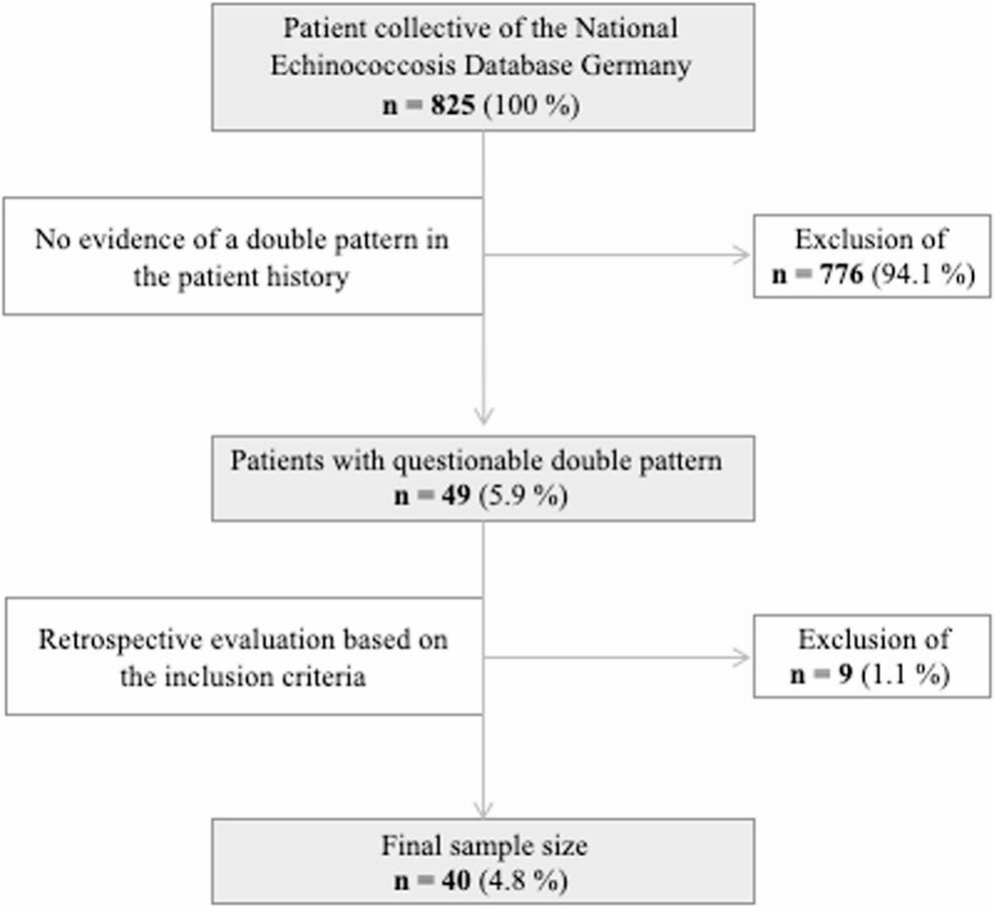
Echinococcosis Multilocularis Ulm Classification-Ultrasound (EMUC-US). The ultrasound pictures shown are from the current patient collective

The Echinococcosis Multilocularis Ulm Classification (EMUC)-US classification, established in 2015, provides a standardized tool for categorizing AE-typical hepatic masses, both in a clinical and scientific context. It comprises five categories. The hailstorm, hemangioma-like and pseudocystic pattern are observed most frequently, whereas ossification pattern and metastasis-like pattern is less common (Fig. 1) (Kratzer et al. 2015).

Recognizing and understanding the sonographic features of AE and ensuring timely treatment are crucial for limiting disease progression and preventing lesions from becoming unresectable (Joos et al. 2023). Furthermore, the high cost, limited availability, and radiation exposure of PET-CT underscore the need for further well-validated prognostic markers for AE (Yangdan et al. 2021). At present, the sonomorphological characteristics of AE do not reliably indicate the course of the disease (Sulima et al. 2019). However, some studies suggest that patterns might change during disease progression, highlighting the need for further research on AE’s ultrasound presentation (Schuhbaur et al. 2021). Intrahepatic heterogeneity of lesion sonomorphology is a frequent finding in hepatic metastases (Kratzer et al. 2022; Joos et al. 2023). Although similar variability has been observed in AE, it has not yet been systematically studied. Previous research on the sonomorphologic appearance of AE lesions has mainly focused on the so-called reference lesion, leaving lesions with differing patterns unexamined. We introduced the term “sonographic double pattern” (SDP), to describe the coexistence of different AE lesions within a single liver. Our study represents the first systematic investigation of SDP lesions in AE, providing insights into their prevalence, pattern combinations, and intrahepatic distribution.

## Methods

### Study collective

We performed a retrospective analysis of clinical and imaging data based on the National Database for Echinococcosis Germany (*n* = 825, as of 08/2023) (Schmidberger et al. 2018). The database manages all patients with AE who have presented to the special outpatient clinic for echinococcosis at Ulm University Hospital since 1992. Inclusion in the database system is voluntary.

**Fig. 2:**
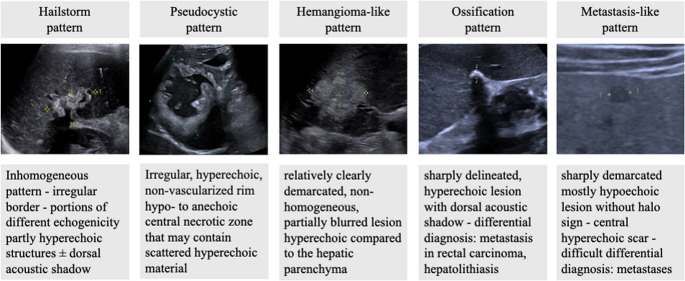
Flowchart illustrating case selection for the current double pattern study based on the patient collective of the National Echinococcosis Database Germany (08/23)

Inclusion criteria for the study were at least two hepatic lesions of different patterns on reference ultrasound. In addition, complete, reproducible documentation of the archived image and video material, was required. The *n* = 49 patients with a questionable sonographic double pattern (SDP) were re-evaluated by two experienced independent sonographers based on the inclusion criteria, whereby 9 patients had to be excluded. Thus, the final number of subjects comprised *n* = 40 patients with a confirmed SDP (Fig. 2).

### Ultrasound examination

All sonographic criteria such as size, number, localisation, pattern and combination of patterns were recorded using the reference US according to the criteria of the EMUC-US classification (Fig. 1) (Kratzer et al. 2015). This was usually the last US examination performed in clinical routine. Inadequate documentation or poor-quality image and video material led to additional, older documented US examinations being used for the current study. In the case of surgical resection of hepatic lesions, the US examination prior to the surgical procedure was analysed. The lesion that showed the largest diameter or a strongly superior number of lesions (metastasis-like pattern) on the first US examination performed in domo was considered the reference lesion. Further intrahepatic lesions that did not correspond to the pattern of the reference lesion were categorised as double pattern lesions. Additional lesions with a third pattern were consequently categorised as triple-pattern lesions. All sonographic criteria were documented accordingly for the additional lesions.

The assignment of the lesions to one of the five sonographic patterns was carried out by three independent examiners (Kratzer et al. 2015). The initial pattern assignment was taken from the written documentation of the physician who conducted the reference US. Subsequently, a retrospective evaluation of all relevant US image and, preferably, video material was conducted by two examiners with extensive experience in the application of the EMUC-US classification. The two investigators were blinded to the initial assessment and acted independently. In addition, they were explicitly instructed to achieve pattern assignment based on the EMUC-US. Any intermediate patterns and patterns that could not be clearly assigned to a category were categorised based on the predominant sonomorphological component. All US examinations were carried out by convex probes (C5-1 MHz, C9-1 MHz) from state-of-the-art US devices (Aplio I800, Siemens S3000, Philips Epiq 7, Philips IU22, GE LOGIQ E9, Samsung RS85).

### Clinical data

Collected clinical data included information on therapy (type, dose, duration), serology, ^18^FDG-PET status and AE case definitions as per Brunetti et al. (2010). Due to the small number of subjects, the case definition ‘possible’ was also included in the analysis. In those three cases, particular attention was paid to ensure AE-typical liver lesions. Patients with positive serology but inconclusive imaging findings were excluded.

### Statistical analysis

For statistical analysis we used the IMB SPSS statistics software (version 29.0.1.0). Descriptive statistics such as absolute and relative frequencies, along with measures of central tendency and dispersion were calculated. Non-normally distributed data was analysed using the Wilcoxon rank-sum test. Pearson’s χ2 and exact fisher tests were employed to assess potential association or independence between two dichotomous variables. All tests were conducted two-sided A significance level of *p* < 0.05 was considered statistically significant with a 5% probability of error.

## Results

### Patient collective

The cohort of 40 patients consisted of 26 (65%) women and 14 (35%) men. According to the current diagnostic criteria of Brunetti, 25 (62.5%) were classified as ‘Probable’ cases, while 12 (30%) were ‘Confirmed’ and only 3 (7.5%) were ‘Possible’ cases. The mean average age at first diagnosis was 51.3 ± 16.2 years, with a range of 20 to 79 years. The mean age at the time of reference US was 59.0 ± 16.4 years, ranging from 20 to 89 years. The inclusion criteria required at least two lesions, 13 (32.5%) patients presented two lesions, 16 (40%) had 3–5 lesions, 6 (15%) showed 6–10 lesions, and 5 (12.5%) had more than 10 lesions.

All patients who were receiving drug therapy up to the time of the reference US (38/40, 95%) were initially given albendazole (ABZ). In the course of the disease, two of the patients undergoing treatment switched to mebendazole. Of the two patients not undergoing drug therapy, one refused, while in the other case, treatment was initiated only after the reference US. At the time of the reference US, only 70% of the patient collective was still undergoing drug therapy (28/40). A total of 5% (2/40) never underwent drug therapy with BMZ, one patient showed absolute ABZ intolerance with severe hepatotoxicity, which led to the termination of therapy. In the remaining 22.5% of patients (9/40), treatment was intentionally discontinued based on suspected inactivity, as indicated by negative PET/CT findings and serological markers (Table 1). No statistically significant correlation was found between the pattern combination and treatment discontinuation.

**Table 1:** Patient characteristics and sonographic aspects

	Frequency (%) mean ± SD
Sex	
Male	26 (65,0)
Female	14 (35,0)
AE case definition	
Confirmed	12 (30,0)
Probable	25 (62,5)
Possible	3 (7,5)
Age at first diagnosis (year)	51,3±16,2
Age at reference US (year)	59,0±16,4
Number of AE lesions	
2	13 (32,5)
3-5	16 (40,0)
6-10	6 (15,0)
>10	5 (12,5)
Pattern of the reference lession	
Hailstorm pattern	28 (70,0)
Metastasis-like pattern	2 (5,0)
Pseudocystic pattern	6 (15,0)
Hemangioma-like pattern	4 (10,0)
Lesion size	54,2±29,7
Hailstorm pattern	49,4±24,5
Metastasis-like pattern	18,5±13,4
Pseudocystic pattern	92,7±33,6
Hemangioma-like pattern	48,5±9,6
Drug therapy	
Initial albendazole	38 (95,0)
No therapy	2 (5,0)
Therapy at the time of the reference US	28 (70)
Intentional treatment discintinuation	9 (22,5)

*SD* standard deviation, *US *ultrasound

## Sonographic characteristics of the reference lesion

### Pattern

According to EMUC-US, 70% (28/40) of the reference lesions presented as the hailstorm pattern, 15% (6/40), as the pseudocystic pattern, 10% (4/40) as the hemangioma-like and 5% (2/40) as the metastasis-like pattern. The ossification pattern was not classified as reference lesion in any of the patients.

### Lesion size

**Fig. 3:**
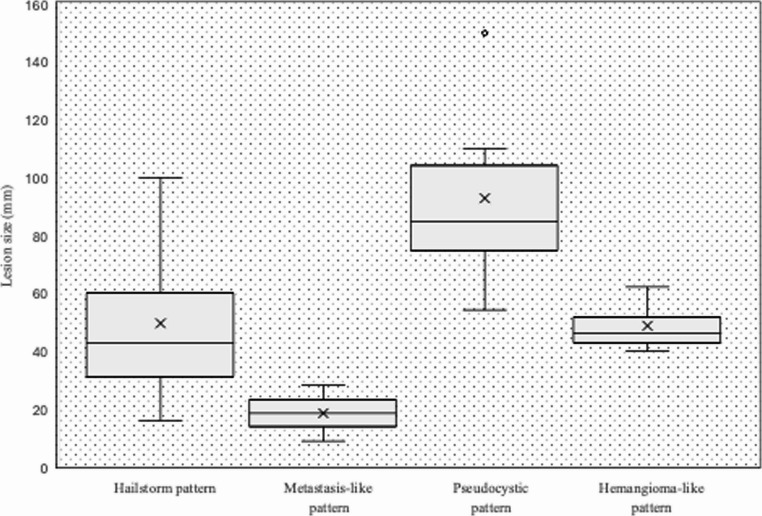
Maximum diameters of the AE reference lesion sorted by sonographic patterns according to EMUC-US

The mean value of the maximum diameter of the reference lesions was 54.2 ± 29.7 mm, with the smallest reference lesion measuring 9 mm and the largest 150 mm. On average, the pseudocystic pattern showed the largest diameters with 92.7 ± 33.6 mm, and the metastatic pattern the smallest with 18.5 mm ± 13.4 mm. The mean lesion size of the hailstorm pattern was 49.4 ± 24.5 mm and that of the hemangioma-like pattern 48.5 ± 9.6 mm (Fig. 3).The study detected statistically significant differences between the lesion sizes of the reference lesions in the pseudocystic pattern compared to the hailstorm pattern (92.7 ± 33.6 mm vs. 49.4 ± 24.5 mm; *p* = 0.006), as well as compared to the hemangioma-like pattern (92.7 ± 33.6 mm vs. 48.5 ± 9.6 mm; *p* = 0.019).

## Double pattern

### Prevalence and characteristics

**Fig. 4:**
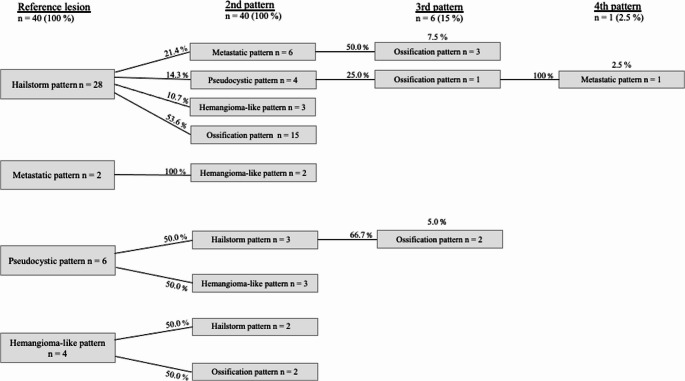
Flowchart illustrating the distribution of pattern combinations starting from the reference lesion

A SDP was found in 40 patients (4.8%) of the national database for echinococcosis (Fig. 4). A total of 34 (85%) patients presented two different patterns, while 5 (12.5%) had a third pattern and one (2.5%) exhibited four different patterns. Further 8 patients (20%) presented lesions in the right lobe only, while 32 (80%) of double patterns were located in both lobes. There was no distribution over the left lobe alone.

In the patient population analysed, ossification patterns occurred in 57.5% of patients, making up 26.4% (23/87) of all evaluated lesions. However, they were not defined as a reference lesion in any case. The occurrence of an ossification pattern was associated with a hailstorm pattern in 91.3% of cases (21/23). The remaining 8.7% of the ossification patterns occurred in combination with the hemangioma-like pattern (2/23). The study revealed statistically significant differences in the frequency of pattern combination of the hemangioma-like pattern and the hailstorm pattern with the ossification pattern (*p* = 0.005).

### Combination of patterns

**Fig. 5:**
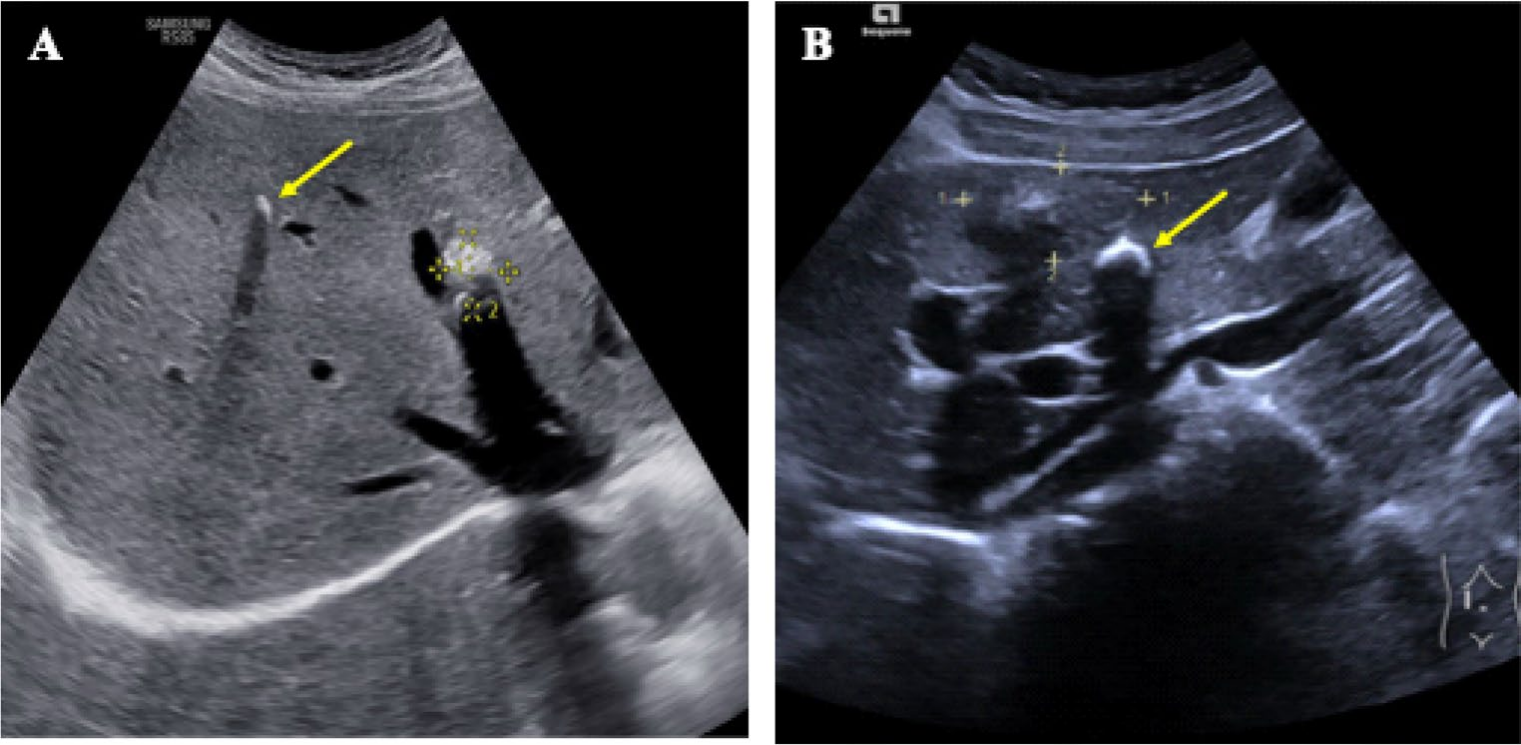
two individual patients (A&B) exhibiting a double pattern consisting of hailstorm pattern (marked) and ossification pattern (arrow)

A total of 10 pattern combinations between two different patterns were found. Thus, every possible combination of two patterns was possible. A total of 21 patients (52.2%) presented both hailstorm pattern and ossification pattern (Fig. 5). The combination of hailstorm/metastasis-like pattern and hailstorm/hemangioma-like pattern each occurred in 7 (17.5%) patients. The constellation of hailstorm pattern/hemangioma-like pattern was represented five times in the examined patient collective (12.5%) (Table 2).

**Table 2:** Overview of the four most common pattern combinations between two patterns

	Frequency (%)
Hailstorm pattern & Ossifiation pattern	21 (52,2)
Hailstorm pattern & Pseudocystic pattern	7 (17,5)
Hailstorm pattern & Metastasis-like pattern	7 (17,5)
Hailstorm pattern & Hemangioma-like pattern	5 (12,5)

### Pattern combinations with more than two patterns

**Fig. 6:**
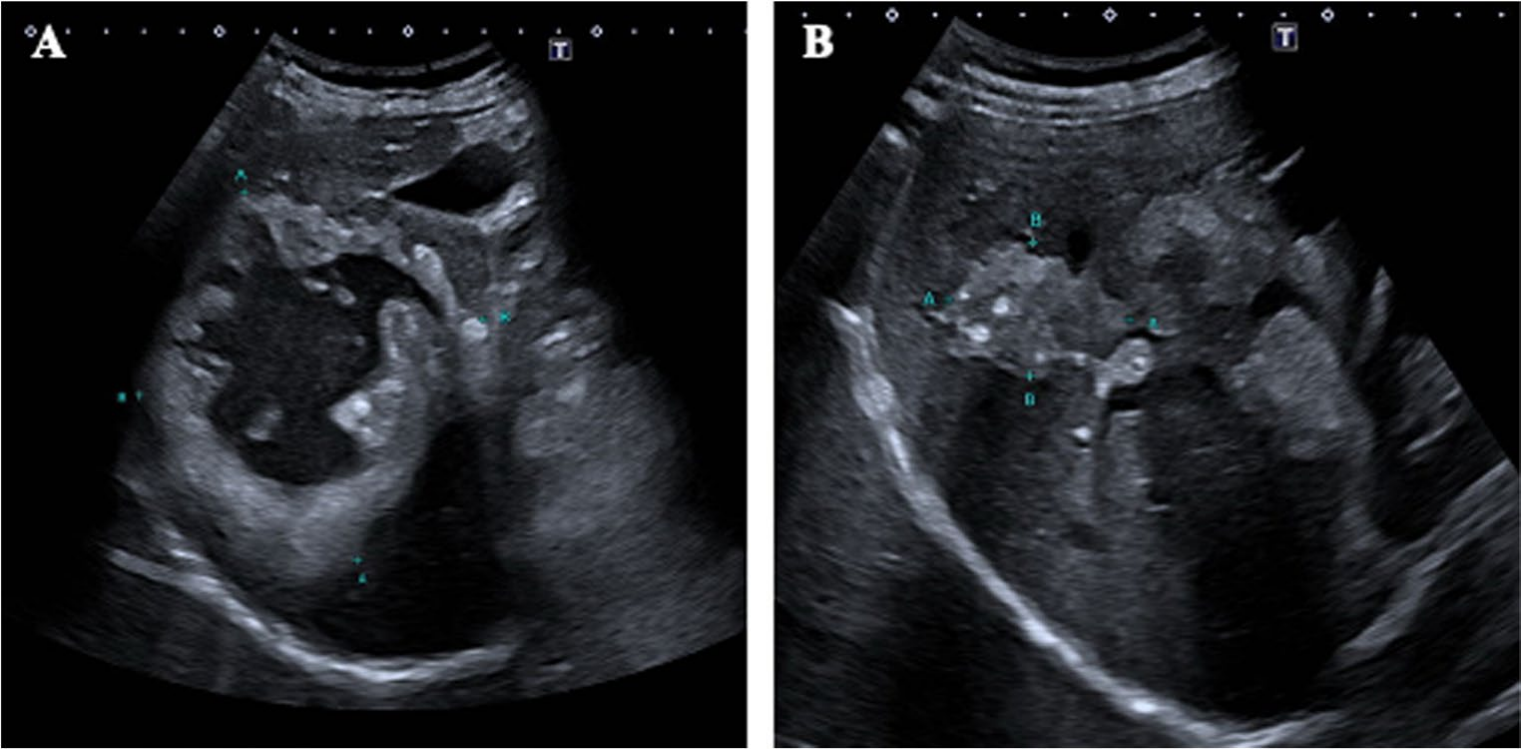
Patient with pseudocystic reference lesion (**A**) with a size of 68 × 63 × 87 mm and double pattern of the hailstorm type (**B**) with a maximum size of 35 × 34 × 24 mm

In all 6 patients who presented with more than two patterns, both the hailstorm and the ossification pattern were involved (Fig. 6). The metastatic pattern occurred in 10% of all patients (4/40) as an additional third pattern. One patient exhibited an additional fourth lesion in the form of a pseudocystic pattern.

### Number of AE lesions according to the pattern

**Fig. 7:**
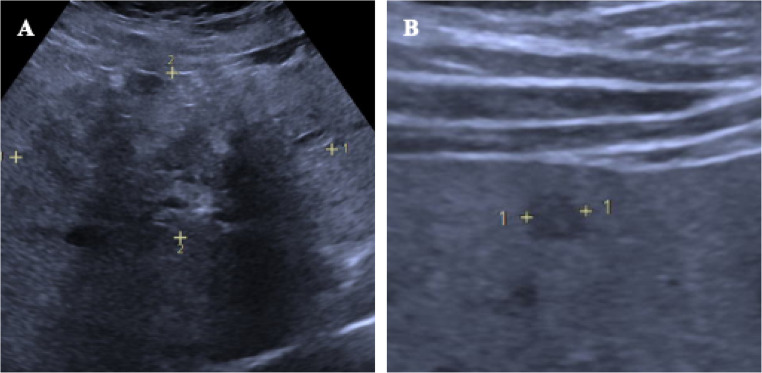
Patient with hailstorm reference lesion (**A**) with a size of 99 × 52 mm and double pattern of the metastatic type (**B**) with a maximum size of 5 mm

A total of 193 lesions were found, averaging 4.8 ± 2.5 lesions per patient, ranging from 2 to over 20 lesions. Analysis of AE reference and double pattern lesions showed a significant difference in lesion number between the metastatic and pseudocystic patterns (unifocal occurrence of the pattern 9/10 vs. 3/9; *p* = 0.0198). The pseudocystic pattern was characterised by a solitary hepatic lesion in 90% of cases (9/10), while the metastatic pattern occurred multifocally in 66.7% of cases (6/9) (Fig. 7). Although the hailstorm and hemangioma-like patterns also appeared more frequently as solitary lesions, this finding was not statistically significant (*p* > 0.05).

## Discussion

### Prevalence of double patterns

The cross-sectional study we conducted was the first to describe the simultaneous growth patterns of AE lesions in US imaging while also quantifying the occurrence of these double patterns and their combinations. Our results emphasize the possibility of a double pattern manifestation of alveolar echinococcosis. With a prevalence of less than 5%, this, however, is a rare phenomenon. As previous studies relate exclusively to the reference lesion, no comparison is possible to date. The observed low prevalence of double pattern lesions may be due to various factors. The occurrence of a solitary AE lesion excludes the formation of a double pattern. Both in the study by Kratzer et. al. and in a study by Tiao-Ying et al. with 311 cases of AE manifestation, solitary AE lesions were the most common (2015, 2004). Multifocal hepatic AE lesions have so far only been assigned to a single pattern in all studies known to us. This may indicate a certain homogeneity in the growth pattern of the lesions. However, these studies relate exclusively to the reference lesion, meaning that possible double patterns were not depicted. As far as we know, the term ‘sonographic double pattern’ (SDP) has been newly defined as a result of this work. Therefore, examiners may be less sensitized to the detection of double patterns. Moreover, smaller, marginal lesions cannot always be adequately recognised by US, leading to possible underestimation of SDP occurrence. Further studies with greater case numbers are needed to confirm our preliminary results and draw conclusions about the overall prevalence of SDP.

### Growth behaviour

Hepatic AE lesions can vary considerably in morphology, size, and number. The great variability in growth behaviour of AE has been described by several studies, tough its cause is not yet fully understood (Calame et al. 2022; Kratzer et al. 2022; Tao et al. 2024; Kratzer 2025). In our study, we observed lesion diameters ranging from 5 mm in double patterns of metastasis-like, hemangioma-like, and ossification types to as large as 15 cm in pseudocystic patterns. Pseudocystic patterns appeared significantly more often as unifocal lesions, whereas metastatic patterns tended to manifest multifocally, with some patients showing over 20 metastatic-type lesions. These findings are consistent with previous studies noting similar trends in lesion size and count across US patterns (Sulima et al. 2019; Schuhbaur et al. 2021). Double pattern lesions add an additional dimension to this hypothesis, as they describe the additional potential of AE lesions to present different expressions within a single liver. To improve the understanding of AE growth behaviour, further studies of SDP in US and other imaging modalities (CT, MRI) are needed.

### Combination of patterns

The evaluation of the patterns according to the EMUC-US classification revealed that AE lesions of different patterns can occur together. Most patients (85%) showed two different patterns, but the occurrence of three (12.5%) and in one case four different patterns was also observed. The manifestation of *E. multilocularis* is therefore not confined to a specific growth pattern and may even take on up to four different forms within a single patient. A total of ten different combinations between two patterns and thus every possible combination could be observed. The hailstorm pattern was involved in the four most frequent pattern combinations. The hailstorm pattern is recognized as the most common manifestation of AE lesions, observed in over 50% of cases according to the study by Kratzer et al. and a study conducted by a Polish research group (2015, 2019). Slightly over half of the patients (52.2%) exhibited a combination of the hailstorm and ossification patterns, making it the most common pattern pairing. Over half of the patients showed a calcification structure consistent with an ossification pattern, which co-occurred with a hailstorm pattern in more than 90% of cases. The hailstorm pattern was significantly more likely to be associated with ossification compared to the hemangioma-like pattern. Both the ossification and hailstorm patterns are defined by varying degrees of calcification, with the ossification pattern being fully calcified and smaller in size, while the hailstorm pattern is larger and only partially calcified. In contrast, the hemangioma-like pattern lacks these calcification features. These findings suggest a tendency for patterns with calcification to co-occur.

### Differential diagnosis

The diagnosis of AE remains challenging due to its variable imaging appearance and the broad spectrum of differential diagnosis. Hepatic AE lesions can resemble various benign and malignant, cystic, or solid liver lesions (Kratzer 2025). In B-scan sonography, metastatic and hemangioma-like patterns pose the greatest challenge. Contrast- enhanced ultrasound (CEUS) plays a key role in differentiation from coincidental preexisting lesions such as classic hepatic hemangioma or true metastases (Philipp et al. 2023, Schweizer et al. 2023). Typical hemangiomas show early arterial ring enhancement with increasing centripetal fill-in (“iris diaphragm phenomenon”) and sustained hyperenhancement in the late phase. In contrast, AE lesions demonstrate bulbous rim enhancement with a complete lack of central contrast uptake in the late phases, known as the “black hole sign” (Schweizer et al. 2023). CEUS can also help in distinguishing AE lesions from true hepatic metastases. Whereas true metastases typically show central contrast uptake with a distinct wash-out phenomenon in late phases, metastasis-like AE completely lacks central enhancement and exhibits the black-hole sign (Schweizer et al. 2023).

### Activity assessment

Since Reuter et al. first described AE in ^18^FDG-PET/CT imaging, PET has remained the only method for assessing the metabolic inflammatory activity of AE lesions (1999). However, it should be noted that the increased ^18^FDG- uptake around parasitic lesions can only be interpreted as an expression of inflammatory immune response (Caoduro et al. 2013). Data from Stumpe et al. emphasize that increased ^18^FDG uptake does not correspond to the vitality of the AE lesion or the parasite (2007). In the future, magnetic resonance imaging may become the method of choice for assessing perilesional AE metabolic activity (Eberhardt et al. 2022).

Since BMZ therapy typically only provides a parasitostatic effect, lifelong, continuous treatment has traditionally been recommended. However, the potential for a parasiticidal effect of BMZ has been discussed for a while. Thus, a discontinuation of treatment can be justified in the case of negative FDG-PET/CT and specific negative serum antibodies (e.g. anti-Em18 or anti-EmII/3–10) (Ammann et al. 2015; Deibel et al. 2022; Husmann et al. 2021). In the present study cohort, treatment was purposefully discontinued in nine patients due to presumed lesion inactivity, supported by negative FDG-PET/CT results and serological markers. No statistically significant correlation was identified between pattern combinations and treatment withdrawal.

The significant heterogeneity in patterns expression can currently not be used as a disease progression parameter (Sulima et al. 2019). The constitution of the patterns and their combination are most likely subject to multifactorial influences. The immunological condition of the host plays a decisive role in the initial expression of hepatic lesions (Deplazes et al. 2005). Small, purely calcified lesions in the form of an ossification pattern are often referred to as ‘died-out lesions’ in CT and most likely represent a metabolically inactive lesion (Deibel et al. 2022; Rausch et al. 1987). This suggests that the increase in calcifications may reflect a pronounced immune response of the host. However, contrary to previous assumptions, this does not necessarily indicate the death of the parasites (Gottstein et al. 2014).

Recently published work indicates, that the sonomorphology of AE lesions may change over time leading to pattern reclassification. In a longitudinal study including 49 patients, a change in pattern was observed in 15.3% of cases (9/59). This change occurred exclusively in lesions initially classified as hemangioma-like or pseudocystic pattern, whereas lesions with a hailstorm or metastatic-like pattern remained stable over time. The most frequent transformation involved an increase in calcified structures, with lesions evolving from a hemangioma-like to a hailstorm pattern (Schuhbaur et al. 2021). Considering that nearly all patients in the study received anthelmintic treatment, the observed increase in calcifications may be influenced by the continuous drug treatment, representing a chronification process. Thus, the varying degrees of calcification in different patterns cannot be used as a reliable marker for the metabolic inactivity of the entire lesion.

The search for suitable non-invasive markers for the assessment of parasite activity continues to be a central topic of AE research. Studies comparing the cost-efficient and radiation-free contrast-enhanced ultrasound examination (CEUS) with the established gold standard for activity assessment, FDG-PET/CT, provide particularly promising results (Tao et al. 2024; Yangdan et al. 2021; Philipp et al. 2023; Schweizer et al. 2023). The value of PET-MRI in activity diagnostics cannot be conclusively assessed yet (Eberhardt et al. 2022). A completely new approach for a deeper understanding of double patterns could be the use of AI systems (Yang et al. 2023).

### Limits of the study

The retrospective design of the study must be seen as a limitation. The rarity of the disease, along with the few cases exhibiting an SDP complicates the assessment of inter-observer reliability. While one of the examiners was a key contributor to the development of the EMUC-US classification, suggesting low intra-observer variability, inter-observer variability remains a potential challenge. Additionally, due to the small sample size and subgroups, small effects may not be validly detected. Further studies with larger case numbers, preferably multicentre and international studies, are necessary to confirm our findings.

## Conclusions

The significance of SDP lesions in hepatic AE is not yet fully understood. Although rare, the simultaneous detection of different sonographic AE manifestations within a patient demonstrates the pronounced heterogeneity of the disease and makes the classification of different stages over time difficult. Currently, the expression of different ultrasound patterns and their combinations are not reliable indicators for monitoring disease progression. Further studies should include double pattern lesions to better understand the highly variable growth behaviour of AE. Comparative control group studies are necessary to draw conclusions about possible correlating factors. Prospective studies with a larger number of subjects could also be useful in this area to capture growth dynamics over time.

## Data Availability

The data sets generated and analyzed as part of the current study are not publicly accessible, as they involve the evaluation of stored image information. The image data is part of an internal findings documentation system that cannot be made accessible in its entirety.

## Abbreviations

AE: Alveolar echinococcosis
US: Ultrasound
EMUC: Echinococcosis Multilocularis Ulm Classification
^18^FDG-PET/CT: ^18^Fluordesoxyglucose positron emission tomography / Computed tomography
PET: Positron emission tomography
BMZ: Benzimidazoles
CEUS: Contrast-enhanced ultrasound
CT: Computed tomography
MRI: Magnetic resonance imaging
SDP: sonographic double pattern
